# Identifying a suitable model for predicting hourly pollutant concentrations by using low-cost microstation data and machine learning

**DOI:** 10.1038/s41598-022-24470-5

**Published:** 2022-11-19

**Authors:** Rongjin Yang, Lizeyan Yin, Xuejie Hao, Lu Liu, Chen Wang, Xiuhong Li, Qiang Liu

**Affiliations:** 1 Chinese Research Academy of Environmental Sciences, Beijing, China; 2 State Key Laboratory of Remote Sensing Science, College of Global Change and Earth System Science, Beijing Normal University, Beijing, China; 3 Higher Institute of Computer Modeling and Their Applications, Clermont Auvergne University, Clermont-Ferrand, France

**Keywords:** Environmental sciences, Environmental social sciences

## Abstract

Accurately predicting the concentration of PM_2.5_ (fine particles with a diameter of 2.5 μm or less) is essential for health risk assessment and formulation of air pollution control strategies. At present, there is also a large amount of air pollution data. How to efficiently mine its hidden features to obtain the future concentration of pollutants is very important for the prevention and control of air pollution. Therefore we build a pollutant prediction model based on Lightweight Gradient Boosting Model (LightGBM) shallow machine learning and Long Short-Term Memory (LSTM) neural network. Firstly, the PM_2.5_ pollutant concentration data of 34 air quality stations in Beijing and the data of 18 weather stations were matched in time and space to obtain an input data set. Subsequently, the input data set was cleaned and preprocessed, and the training set was obtained by methods such as input feature extraction, input factor normalization, and data outlier processing. The hourly PM_2.5_ concentration value prediction was achieved in accordance with experiments conducted with the hourly PM_2.5_ data of Beijing from January 1, 2018 to October 1, 2020. Ultimately, the optimal hourly series prediction results were obtained after model comparisons. Through the comparison of these two models, it is found that the RMSE predicted by LSTM model for each pollutant is nearly 50% lower than that of LightGBM, and is more consistent with the fitting curve between the actual observations. The exploration of the input step size of LSTM model found that the accuracy of 3-h input data was higher than that of 12-h input data. It can be used for the management and decision-making of environmental protection departments and the formulation of preventive measures for emergency pollution incidents.

## Introduction

PM_2.5_ refers to the particulate matter with an aerodynamic equivalent diameter less than or equal to 2.5 μm in the ambient air^1^, which is the main monitoring object of the National Air Quality Monitoring Station. It is the pollutant produced by human production and life that exceeds its own purification capacity and may have an impact on the environment^2^. PM_2.5_ is a category which covers a broad range of pollutants, including those produced by human activities, those produced by natural processes (e.g., desert dust), and as the result of chemical and physical processes in the atmosphere (e.g., molecules aggregating together to form particles). The effects of PM_2.5_ on human health have been extensively studied^3^. No matter short-term outbreak or long-term accumulation of this pollutant, it will have an important impact on mankind. In particular, the smog caused by PM_2.5_ not only makes the weather cloudy and with low visibility, which will cause greater hidden dangers to people’s travel safety^4^, but also increases the mortality rate of diseases related to the respiratory, cardiovascular, and nervous systems^5,6^. In addition, localized air pollution may also have an impact on regional and even global climate change^7^, and then may cause other environmental and health problems^8,9^. Therefore, it has become a global consensus that the monitoring and prediction of PM_2.5_ pollutants are extremely important.

As the capital of China, Beijing is densely populated and seriously affected by air pollution. The frequent outbreak of severe weather phenomena such as haze and sandstorm and the accompanying increase of respiratory diseases are particularly urgent for such a first tier city with economic development and dense population. Therefore, its air pollution problem has become the focus of people's attention.Therefore, it is necessary to use machine learning method to predict PM_2.5_ concentration. Yan Xing et al. Improved the precision of PM_2.5_ concentration inversion based on MODIS sensor by using the deep learning network. The spatial–temporal distribution characteristics of PM_2.5_in Beijing were obtained through the analysis of the retrieved high spatial–temporal PM_2.5_ results^45^.The spatial distribution trend of pollutants depends on accurate ground station data.

Air pollution prediction research has experienced a development process from qualitative analysis to quantitative modeling from the 1960s to the present. In 1960, Lawrence E qualitatively described the characteristics of weather conditions such as wind direction and atmospheric stability under the condition of poor air quality data, and speculated that the high incidence period of air pollution could be estimated based on the prediction of weather conditions. Although there was no quantitative equation, this exploration laid a theoretical foundation for the subsequent emergence of quantitative analysis models, especially numerical prediction models^10^.

Time series prediction analysis is a mathematical method to reason about the performance results of the upcoming periods based on all the laws and characteristics of past materials and data^11^, which has been widely used in various fields, including the economic market^12^, energy consumption^13^, biomedicine^14^, environmental monitoring^15^. According to the principle of model construction, the time series prediction models of air pollutants are divided into two types: mechanism models and non-mechanism models.

The mechanism model simulates the transformation and diffusion processes of pollutants in the air based on atmospheric dynamics. The movement of pollutants in the horizontal and vertical directions, the emission of different pollution sources, and the physical and chemical properties evolutions of pollutants in the air are fully considered. Commonly used mechanism models include Nested Air Quality Prediction Modeling System (NAQPMS) and City Air Pollution Numerical prediction System (CAPPS) independently developed by China, as well as the world-widely used CMAQ^16,17^, CAMx^18^, WRF- Chem^19^, ADMS^20^, CHIMERE et al.^21^. The prediction system in China can not only predict the pollutants of a single element such as PM_2.5_, SO_2_, O_3_, et al. on a regional scale^22^, but also simulate the occurrence of pollution. Wu Ying et al. analyzed the prediction effects of Ozone in Taizhou via the NAQPMS and CMAQ models and found that the performance of the two models in different seasons has their own advantages and disadvantages, and the overall prediction effects are both within the ideal range^23^. Ma Siqi et al. used four models of WRF-Chem, CHIMERE, CMAQ and CAMx to simulate the sandstorm weather in Northeast China and obtained the performance effects of each model under different parameter configurations. Although there are slight differences between the predicted results of each model and the observed PM_10_ (Inhalable Particles, usually refers to particles with a particle size below 10 microns) concentration, each model has relatively truly restored the occurrence process of sandstorm^24^. Taking more comprehensive factors into account, mechanism models express the entire process of pollutant generation, transportation, transformation and dissipation by a parameterized equation, which is more in line with the actual emission situation. However, it is necessary to consider factors such as complex and changeable meteorological fields, pollutant emission inventories, and geographical features when constructing a numerical forecast model. Thus, model construction is difficult for people who do not have the knowledge of traditional meteorology. Furthermore, due to simplification effects, lack of parameters or unrepresentative observations, it may not be possible to simulate atmospheric diffusion under stable conditions, which usually results in low prediction accuracy^25^^.^

The prediction of the non-mechanism model does not require complex parameters and accurate physical and chemical equations. It is dedicated to better prediction results without considering the mechanism process. Through statistical learning of massive historical pollutant data, it summarizes the law of concentration changes and predicts the pollutant concentration for a period in the future. Commonly used statistical models include generalized linear regression (LR), autoregressive integrated moving average (ARIMA), projection tracking model (PP), principal component analysis (PCA), support vector regression (SVR), et al.^26^, all of which realize the function of prediction by establishing linear regression relationship between input time series pollutant data and output results. These models have also achieved good results in some research. Zhang Yuli et al. constructed a power function multiple linear regression model of PM_2.5_ in Shanghai. After cross-validation, the correlation coefficient was concluded as 0.94, the root means square error as 1. Moreover, since the fitting relationship between the predicted result graph and the true value was good, it can be used as the prediction model under the ideal state and provides relevant control recommendations to the local government^27^. Peng Sijun et al. conducted the prediction in the ARIMA model by using Wuhan's PM_2.5_ daily average concentration data. Comparing with the results of the gray model, they obtained the better effect of the segmented time series prediction in the short-term PM_2.5_ prediction^28^. Bing-Chun Liu et al. carried out a collaborative prediction on the Air Quality Index (AQI) in the Beijing-Tianjin-Hebei region in the SVR model, and found that the MAPE (The mean absolute percentage error. It is the descriptive accuracy. Because the mean absolute percentage error itself is often used as a statistical measure of forecast accuracy, such as time series forecasting) in all cases was between 0.05 and 0.09, which means the prediction results are highly reliable^29^. Although they have performed well in the prediction of air pollutants, they still have some shortcomings compared with other nonlinear technologies^30^. Because the pollutant time series are not simply linear relationships, the influence of other factors such as wind speed, wind direction, and human activities are also involved. Comparing the pros and cons of linear and non-linear methods in predicting the concentration of PM_10_, Abdullah, S. et al. concluded that the error range of the non-linear model in predicting the concentration of particulate matter was reduced by at least 30%, no matter in rural, suburban or urban areas. Meanwhile, the artificial neural network can generate more accurate data of PM_10_^31^. Thus, scholars have mostly focused on non-linear models in the study of predictive models in recent years.

Machine learning (ML), as an intelligent learning method that integrates multidisciplinary knowledge and uses computers to simulate human activities^32^, gives full play to its advantages in fitting non-linear problems, especially its ability to automatically classify and identify and efficiently process and analyze data in the current era of big data. Decision trees, random forests, Bayesian learning, artificial neural networks, et al. are all core algorithms of machine learning, which have been applied to air pollution prediction research by many scholars at home and abroad. The first several algorithms were used in the prediction of air pollutants earlier because of their relatively simple structure and easy implementation. Gocheva-Ilieva et al. proposed a general method to establish a nonlinear model of environmental time series quality by using the powerful data mining technology of Classification and Regression Tree (CART), and the results are in good agreement with the measured data. CART is better than ARIMA^33^ in predicting the concentration of PM_10_ in Europe. Ren Cairong and Xie Gang predicted the PM_2.5_ concentration in Taiyuan based on random forests and meteorological data. Model verification showed that random forests model has better accuracy and recall rate^34^. Sujit K. Sahu et al. came up with a Bayesian hierarchical space–time model to predict Ozone concentration in the eastern United States, and found that the data obtained by the new model was more accurate than the model results based on only Eta-CMAQ prediction data. The time resolution was improved, and the prediction of the concentration value in the space position was more accurate^35^. Osowski, Stanislaw and Garanty, Konrad predicted atmospheric pollution days in northern Poland in the methods of support vector machines and wavelet decomposition, and found that the prediction results were in good agreement with the actual measured values, no matter the pollutant type was NO_2_, CO, SO_2_ or dust^36^.

Compared with the above-mentioned machine learning algorithms, artificial neural networks have the characteristics of strong fault tolerance and dynamic stability^37^, that is, the requirements for input data are relatively low, which does not have to be continuous and perform smoothly on external influences. Artificial neural networks contain a large number of nodes, consisting of an input layer, an output layer, and at least one hidden layer. As a result, this model can perform highly complex mappings on nonlinear data, thereby inferring the subtle relationship between the input data set and the output parameters. At present, artificial neural networks have many model classifications, including feedforward neural network (FNN), back propagation (BP) algorithm, recurrent neural network (RNN), et al.^38^. With fast calculation speed and high prediction accuracy, they have been widely used in the field of air quality prediction and have achieved good results in the past few years. He Jianjun et al. used meteorological data, pollution emission data, circulation type data derived from WRF model and observation data to derive an ANN model to predict the daily concentration of SO_2_, NO_2_ and PM_10_ in Lanzhou, China. The results showed that the models can reproduce the pollution level and its daily changes well, and the correlation coefficients of the daily averages of the three pollutants ranged from 0.71 to 0.83^39^. Zhang Hong et al. used a BP neural network model with different air quality parameters to predict the temporal and spatial distribution of the annual average concentration of PM_10_ in Taiyuan. The prediction results of the model were consistent with the change trend of the observed value, and the correlation coefficient was 0.72^40^. Mohammad adopted a combination of ANN and Monte Carlo. Taking Tehran as a case, wind speed, temperature, relative humidity and wind direction were selected as the input variables of neural network models to simulate the concentration of five pollutants. The determination coefficient (R^2^) of simulated and observed carbon monoxide, nitrogen oxide, nitrogen dioxide, nitric oxide and PM_10_ pollutant levels is greater than 0.82, showing a high correlation, which also indicates that the method combined with ANNs and MCSs has a good application prospect in analyzing the uncertainty of air pollution prediction^41^.Grivas, G, et al. built a neural network model for hourly concentration prediction of PM_10_ in Athens, and the results were quite satisfactory. The R^2^ of the four-point independent test set was between 0.50 and 0.67, the value of the consistency index was between 0.80 and 0.89. Compared with the multiple linear regression model developed at the same time (R^2^ was between 0.29 and 0.35), the performance of the studied neural network model was superior^42^.

With the deepening of studies, a type of model that can explore the context of time series was introduced into the prediction of atmospheric pollutant concentration. Kangil Kim et al. applied the recursive network LSTM with memory structure to environmental time series problems, such as water pollution, air pollution and Ozone alarm. It turned out that the recursive network with memory had better predictive performance in non-stationary environments and long-term time lag conditions^43^. Yi-Ting Tsai et al. proposed to predict the concentration of PM_2.5_ based on LSTM, and conducted an evaluation experiment of hourly PM_2.5_ concentration prediction at 66 stations in Taiwan, the results of which proved that this method can effectively predict the value of PM_2.5_^44^.

In summary, shallow machine learning, such as decision trees, can be used to predict the concentration of air pollutants, and the prediction performance of the CART algorithm has been evaluated. LightGBM, which is also a decision tree model, has similar results to neural networks when processing massive data features, with fast processing speed and less memory. As a kind of neural network algorithm, the LSTM model has also made certain progress in the prediction of single pollutants such as PM_2.5_.

However, the comparison between the multiple pollutants prediction results of machine learning and neural network in the Beijing area is not clear. This research has conducted in-depth exploration and experiments in order to find the optimal time prediction model.

## Materials and methods

### Data collection

#### PM_2.5_ Data

The PM_2.5_ monitoring data selected in this study are hourly data of 34 stations (There are 35 original stations, but the data of the Botanical Garden Station is discarded due to serious lack of data in 2019 and 2020) on the webstation of Beijing Municipal Ecological Environment Monitoring Center (http://zx.bjmemc.com.cn/?timestamp = 1613378868776), from January 1, 2018 to October 1, 2020. The detailed information of the monitoring stations is shown in Table 1, and the unit is micrograms/cubic meter.

**Table 1:** Informatica of air pollution monitoring station.

ID	Station	Longitude	Latitude	Sort	ID	Station	Longitude	Latitude	Sort
1	Fangshan	116.136°E	39.742°N	Suburb	19	Dongsi	116.417°E	39.929°N	Main Urban
2	Daxing	116.404°E	39.718°N		20	Tiantan	116.407°E	39.886°N	
3	Yizhuang	116.506°E	39.795°N		21	Guanyuan	116.339°E	39.929°N	
4	Tongzhou	116.663°E	39.886°N		22	Flower nishinomiya	116.352°E	39.878°N	
5	Shunyi	116.655°E	40.127°N		23	Olympic Sports Cente	116.397°E	39.982°N	
6	Changping	116.23°E	40.217°N		24	Agriculture exhibition center	116.461°E	39.937°N	
7	Mentougou	116.106°E	39.937°N		25	Wanliu	116.287°E	39.987°N	
8	Pinggu	117.1°E	40.143°N		26	Northern New District	116.174°E	40.09°N	
9	Huairou	116.628°E	40.328°N		27	Fengtai garden	116.279°E	39.863°N	
10	Miyun	116.832°E	40.37°N		28	Yungang	116.146°E	39.824°N	
11	Yanqing	115.972°E	40.453°N		29	Ancient city	116.184°E	39.914°N	
12	Dingling	116.22°E	40.292°N	Control Area (CA)	30	Qianmen	116.395°E	39.899°N	Traffic Pollution (TP )
13	Badaling	115.988°E	40.365°N		31	Yongdingmen Inner	116.394°E	39.876°N	
14	Miyun Reservoir	116.911°E	40.499°N		32	Xizhimen north	116.349°E	39.954°N	
15	Donggao Village	117.12°E	40.1°N		33	South third ring road	116.368°E	39.856°N	
16	Yongledian	116.783°E	39.712°N		34	East fourth ring	116.483°E	39.939°N	
17	Yufa	116.3°E	39.52°N						
18	Liulihe	116°E	39.58°N						

#### Meteorological data

The concentration of PM_2.5_ pollutants is closely related to meteorological parameters. When the weather conditions such as wind speed and temperature are not easy to spread, the concentration of pollutants is greater. Therefore, this study obtained a total of 18 hourly monitoring stations on the ground in Beijing from the National Meteorological Science Data Center (http://data.cma.cn) and matched them with the latitude and longitude of the air pollution monitoring stations. The latitude and longitude information of the 18 stations is shown in Table 2 below. There are 6 data elements, namely 2-min average wind direction (WIN_D, unit: degree), 2-min average wind speed (WIN_S, unit: m/s), temperature (tem, unit: °C), relative humidity (RHU, unit: percentage), precipitation (PRE_1h, unit: millimeter), horizontal visibility (visibility, unit: meter). The 999999 or null in the meteorological monitoring data represent lack of observation due to factors such as monitoring equipment problems, network transmission, server storage, et al. 9999998 represents no observations. 999990 in the rainfall data represents a small amount of rainfall and 999017 in the wind direction data represents a quiet wind. These data values, as a kind of marker, are significantly higher than the normal monitoring data and need to be standardized.

**Table 2:** Information of meteorological monitoring stations in Beijing.

Station	Name	Longitude	Latitude	Station	Name	Longitude	Latitude
54,398	Shunyi	116.37°E	40.08°N	54,499	Changping	116.13°E	40.13°N
54,399	Haidian	116.17°E	39.59°N	54,501	Zhaitang	115.41°E	39.58°N
54,406	Yanqing	115.58°E	40.27°N	54,505	Mentougou	116.06°E	39.56°N
54,416	Miyun	116.52°E	40.23°N	54,511	Beijing	116.28°E	39.48°N
54,419	Huairou	116.38°E	40.22°N	54,513	Shijingshan	116.12°E	39.57°N
54,421	Miyun Shangdianqi	117.07°E	40.39°N	54,514	Fengtai	116.15°E	39.52°N
54,424	Pinggu	117.07°E	40.1°N	54,594	Daxing	116.21°E	39.43°N
54,431	Tongzhou	116.38°E	39.55°N	54,596	Fangshan	116.12°E	39.46°N
54,433	Chaoyang	116.3°E	39.57°N	54,597	Xiayunling	115.44°E	39.44°N

### LightGBM and LSTM

As a neural network algorithm that can memorize sequence information, LSTM is the most widely used in time series forecasting. However, it is mostly a prediction attempt at a single site, and it does not make all predictions for multiple pollutants at all sites in the city. As an improved framework for shallow machine learning decision trees, LightGBM has similar effects to neural network algorithms in terms of processing speed and memory footprint. It is widely used in competitions such as search ranking and CTR prediction and is not currently used for air pollution related predictions.

#### LightGBM

The LightGBM algorithm uses a histogram-based feature ranking method. It divides continuous attribute features into discrete square columns, which reduces the Block structure and computational cost for storage compared with Pre-Sorted. LightGBM is another implementation framework of GBDT^46^, which is a more powerful algorithm that is more suitable for processing big data features. Compared with the XGBoost^47^ algorithm, the decision tree growth strategy used by LightGBM is the Leaf-wise method with depth restriction. The leaves of the same layer are not directly split, but the one with the largest gain is directly split. If the gain is small, the leaf node is not operated. In this way, a decision tree is finally formed. The results of the Leaf-wise strategy generate deeper trees with the same number of splits, and the loss function values are closer to the residuals. However, it is also prone to overfitting. In order to prevent this, the maximum depth is set.

The LightGBM algorithm is based on container features when calculating the gain after segmentation. Compared with the XGBoost algorithm performed on a single data feature, it runs faster and has a cache optimization function.

**Figure 1:**
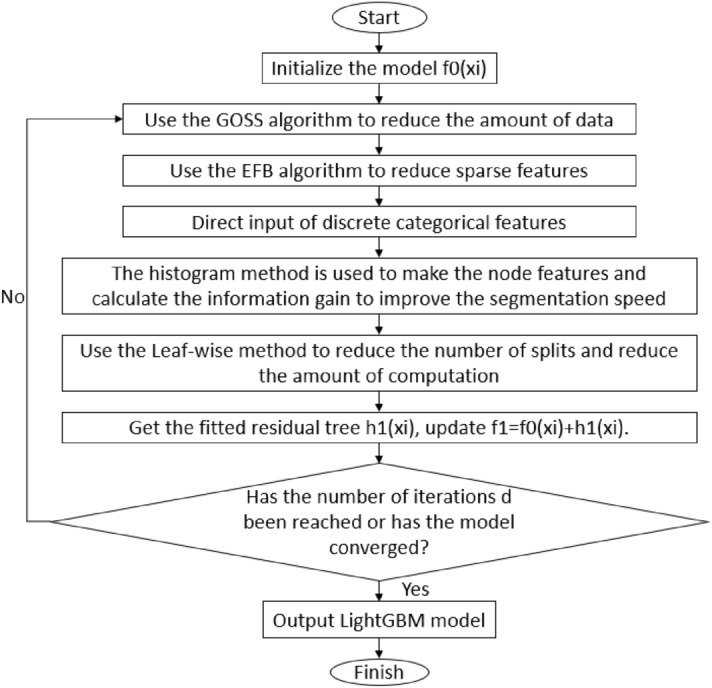
The LightGBM generation process.

LightGBM solves the problem shared by GBDT and XGBoost that only by traversing all samples can the information gain be calculated to find the optimal division point. This problem makes the scalability and efficiency of the latter two algorithms unsatisfactory in massive data processing or high-latitude feature calculation. LightGBM combines one-sided gradient sampling (GOSS) and mutually exclusive feature binding (EFB) algorithms to reduce the amount of data and features, and to ensure the accuracy of regression. The LightGBM generation process is shown in Fig. 1:

#### LSTM

As the core algorithm of machine learning, neural network algorithm is mainly divided into three categories: feedforward neural network, feedback neural network and graph network. The first two algorithms belong to the hierarchical network structure, and the latter one belongs to the interconnected network structure. Like BP, FNN, and CNN for image classification, they all belong to feedforward neural networks, and the information of feedback neural networks can be bidirectional, unidirectional, and self-circulating. This means that it can receive input from neurons in previous layers as well as cyclic feedback from its own nodes, such as Recurrent Neural Networks (RNNs) and Hopfieid Networks.

**Figure 2:**
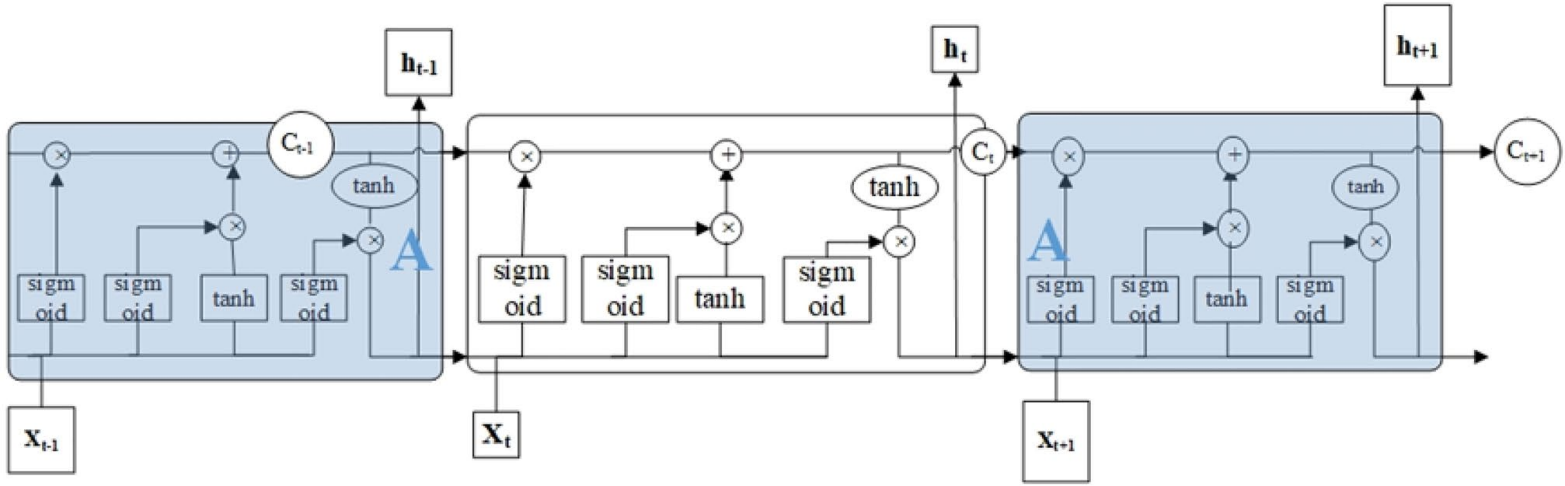
Schematic diagram of the LSTM structure.

Among them, RNN will have gradient disappearance and explosion problems, and Hochreiter and Schmidhuber proposed LSTM network. LSTM is an improved algorithm based on RNN that can store long-term data information. It adds three gates to control the choice of information based on the original structure of RNN. At the same time, a Cell state is added as a "long-term memory" throughout the entire sequence, and its structure diagram is shown in Fig. 2.

## Summary of the data

Before conducting the time series prediction, we first analyzed the air pollution distribution in the study area to understand the trends and causes of changes in air pollution in Beijing in recent years and provide a basis for determining the input factors for time series prediction. This section mainly focused on analysis of the stations.

First, all the obtained hourly data was read and merged, placed in the same file, and then the format was converted into a table with time and station as rows and concentration of PM_2.5_ as columns. On this basis, the average results of different time scales are obtained. According to the classification of monitoring stations, the PM_2.5_ average values of four types, namely main urban areas, suburbs, traffic pollution points, and control area points were obtained. In addition, the concentration of PM_2.5_ was analyzed in the time series of year, season and day (March–May is spring, June–August is summer, September–November is autumn, and December-January is winter.), which will be expanded separately as below.

**Figure 3:**
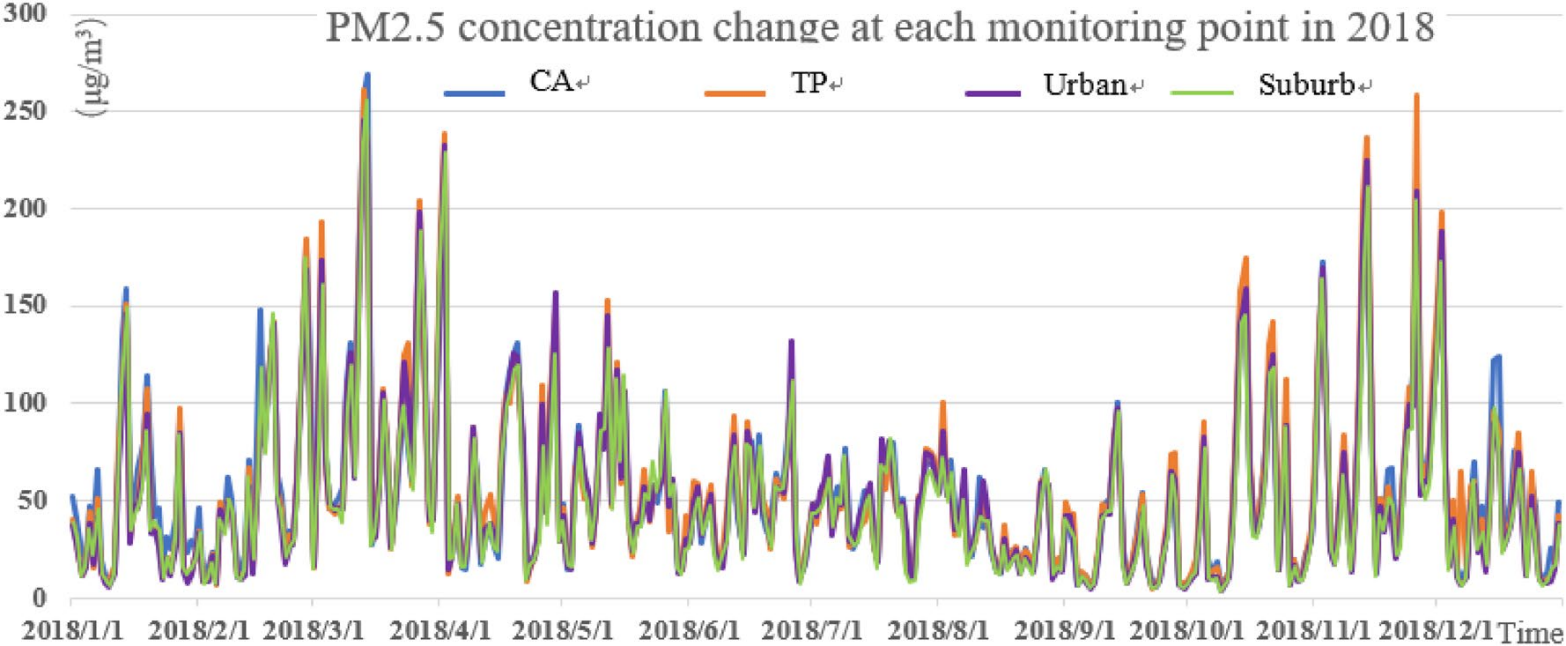
PM_2.5_ concentration change at each monitoring point in 2018.

**Figure 4:**
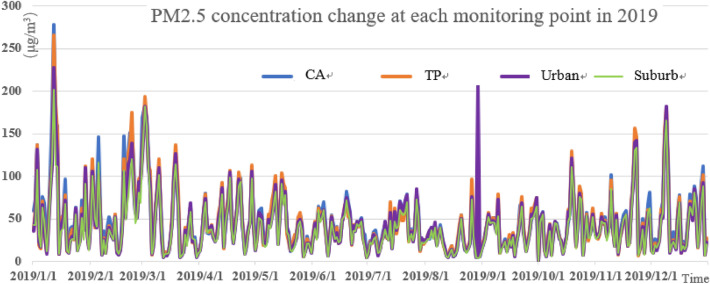
PM_2.5_ concentration changes at each monitoring point in 2019.

**Figure 5:**
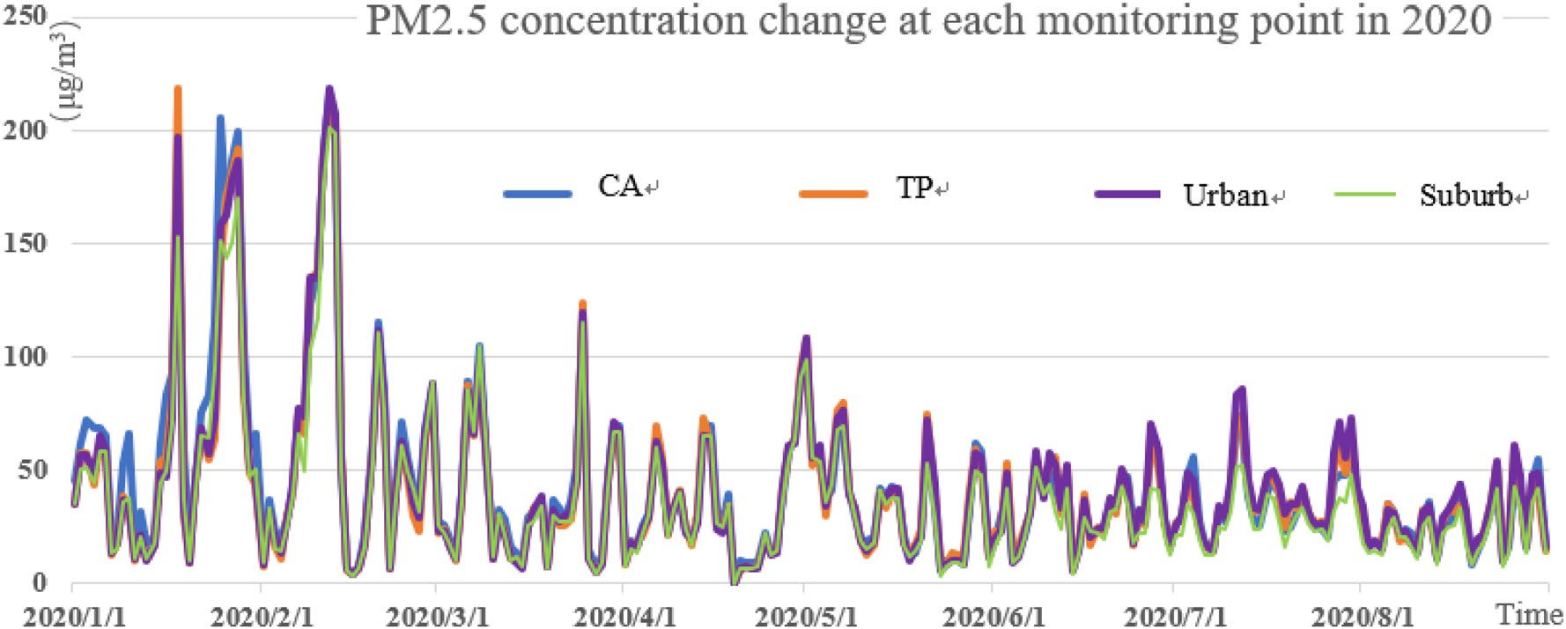
PM_2.5_ concentration changes at all monitoring points in 2020.

It can be seen from Figs. 3, 4, and 5 that the pollution peak of PM_2.5_ concentration was 261.5 μg/m^3^ in 2018, 277 μg/m^3^ in 2019 and 218 μg/m^3^ in 2020. The peak value was reduced by one pollution level, and no serious pollution occurred. The seasonal variation is characterized by that severe pollution occurred in winter and spring, and the concentration of pollutants in summer was the smallest in the year. The winter averages of 2018 and 2019 were 55.71 μg/m^3^ and 59.78 μg/m^3^, respectively, and the summer averages of 2018, 2019 and 2020 were 43.09 μg/m^3^, 33.72 μg/m^3^, and 31.31 μg/m^3^. Especially in the summer of 2020, the daily value was mostly 75 μg/m^3^ and below, and the emission of PM_2.5_ met the good standards of air quality.

The concentration of PM_2.5_ at different stations was different. The number of days in heavy pollution (150 μg/m^3^) and above is shown in Table 3 below. Compared with 2018, the number of days with severely polluted in 2019 decreased nearly a half. The number of pollution days in traffic pollution stations in 2018 and 2019 was much higher than that in the suburbs. In addition, the average value of suburban areas was also the lowest (Table 4). The number of pollution days at each monitoring station in 2020 was very low. In particular, the number of pollution days at traffic pollution stations dropped the most, compared to the previous two years, which may be related to the control of the COVID-19 and home office. According to statistics, the average value of PM_2.5_ was 52.96 μg/m^3^ in 2018 and 44.46 μg/m^3^ in 2019. The decrease was almost the same as the AQI, which was at about 15%, indicating that the PM_2.5_ control measures in Beijing and surrounding areas were effective and had already played a preliminary effect.

**Table 3:** Days of heavy PM_2.5_ pollution in recent three years.

Year	Reference point (day)	Traffic pollution spot (day)	The main (day)	Suburban (day)	Total (day)
2018	15	18	15	12	20
2019	9	9	6	4	12
2020 (As of August 31)	8	7	8	7	8

**Table 4:** The annual average value of PM_2.5_ at each classified monitoring point.

Year	Reference point	Traffic pollution spot	The main	suburban
2018	55.12	54.98	51.83	49.898
2019	47.72	46.00	43.80	40.33

### Proposed PM_2.5_ predictor

#### Classification of data set

The pre-processed and specially selected hourly data from January 1, 2018 to October 1, 2020 are divided into three categories for training, validating, and testing. The data from 2018 to June 30, 2019 is the training set, the data from July 1 to December 31, 2019 is the validation set, and the hourly data from 2020 to August 31 is the testing set. The data of the training and validation set is divided into input factors and output factors. The input factors include 6 meteorological parameters and 7 time characteristic parameters (holidays, working days, weekends, the first day of working days, the last day of working days, the first day of rest days and the last days of rest days). The output factor is the pollutant concentration. The test data set only includes 13 input factors. The predicted output result is the corresponding pollutant concentration.

Although same as the input factors, the difference in the quantity level between the meteorological parameters was relatively large, especially the quantity of the visibility was five digits, while that the wind speed was single digits. Since data of different dimensions participating in the training at the same time may affect the final prediction result, in order to verify the degree of this effect, the data was normalized.

#### Selection of error index

The selection of error index depended on different target tasks of LightGBM. For the regression task of this study, there were multiple choices, such as generally mean absolute error (MAE), mean square error (MSE), RMSE. RMSE prescribes the square root of MSE. With the same data dimension as our training data, RMSE can better describe data characteristics, and was generally used for machine learning model result evaluation. In this study, MAE and MSE were selected as the evaluation indicators of the loss function during the iterative process of the test set and the validation set, and RMSE was used for the final evaluation of the prediction results.

#### Adjust the parameters

There were many parameters of LightGBM. According to the function of the parameters, the parameters were adjusted in the following four steps.

First, the learning rate was determined. The second step was to modify the two parameters to improve the accuracy, namely the maximum depth of the tree and the number of leaf nodes, which together determined the complexity of the decision tree. The third step was to prevent over-fitting. The growth strategy of LightGBM made the tree converge faster, but it also increased the probability of overfitting. In the last step, in order to further improve the accuracy, the original learning rate was reduced to 0.01, 0.03, 0.005, et al. to calculate the RMSE result scores in turn. Finally, the model parameters for training with all the station data were determined as shown in Table 5 below, and the model parameters of a single station were debugged in the same way.

**Table 5:** Key parameter Settings of LightGBM prediction model.

Parameter	Parameter value list	Name of parameter	Parameter value list
num_boost_round	2663	max_depth	12
num_leaves	800	min_data_in_leaf	1
boosting_type	gbdt	bagging_fraction	0.9
learning_rate	0.005	feature_fraction	0.8
metric	Loss function (‘l1’, ‘l2’)	bagging_freq	1

#### Prediction of test data set

After the parameter settings, the above parameters were used for formal model training and validation, through which, the final decision tree model will be determined. Ultimately, the test data set was substituted for prediction to show the results of pollutant concentration in the future.

#### Denormalization

If the pollutant concentration was normalized during the test, the predicted data obtained would be also between 0 and 1. Therefore, it was necessary to restore the data to the original range. Suppose the predicted data is X_1, the minimum value (Min) of the original data column that 0 corresponds to and the maximum value (MAX) of the original data column which 1 corresponds to need to be firstly found, and then the original data would be restored via the function:

1 $$X = X_{1} \left( {{\text{Max}} - {\text{Min}}} \right) + {\text{Min}}$$

The predicted maximum and minimum values of PM_2.5_ are replaced by the maximum and minimum values of PM_2.5_ in the original training data. Similarly, the predicted value range of PM_10_ and O_3_ were restored by replacing the maximum value of the training data.

Among them, the division of the data set, the selection of error index, and the normalization and de-normalization of the data were consistent with LightGBM. The additional processing parts of LSTM will be mainly introduced in the following part.

##### Processing of data set

Since LSTM required the input data to be a three-dimensional tensor, it was necessary to resample the input data to three-dimensional after the data set was classified and normalized. Before being converted into three dimensions, the data need to be converted into time-arranged supervision data, for LSTM relied on time series information. In the training process, the historical pollutant concentration data was involved. If the conversion was not performed, the future value would appear during the training process, so that the prediction model construction will not be correct. We took the following data as example to show the conversion process of supervision data. The data of 3 h including 16 features, namely the pollution concentration factor of the past three moments (including the current moment) and 13 future moments of meteorological and time features was input, and then the output data was the pollutant data of one hour in the future. The process was subsequently demonstrated. First, we marked the original data as time t, insert the first blank line at the top of the original data base as time t − 1, the second blank line as time t − 2, and a blank line at the bottom of the original data as t + 1 time. Then, we merged the four time columns into one table data, deleting the rows with null values. Afterwards, we obtained the final row of data as the supervised time series data. After that, we used the drop function to delete the meteorological and time features of the data at t − 1, t − 2 and t, and added the meteorological and time features at the next moment as input data, and the pollutant concentration at time t + 1 as the label item, the sequence data conversion process was completed.

The data dimension needs to be converted according to the number of samples, the input time and the features. For example, the original size of the PM_2.5_ data table of the Olympic Sports Center Station was (1752042). After the conversion, it became (17520,3,14), where 17520 was the number of samples, 3 was the input time, and 14 was the feature contained in the data at a time.

##### Construction of prediction models

The first step was to define the network, where three layers were set up. The input layer of the LSTM neural network had 64 neurons. The input size was 3 input time steps and 14 input features, which passed the result of each time step to the hidden layer. The LSTM hidden layer also set up 64 neurons, and only output the result of the last time step to the output layer. There was 1 neuron in the fully connected output layer, using a linear activation function.

Secondly, the network was compiled, with default configuration as parameters, MSE as the loss function, and ADAM as the optimization algorithm.

**Figure 6:**
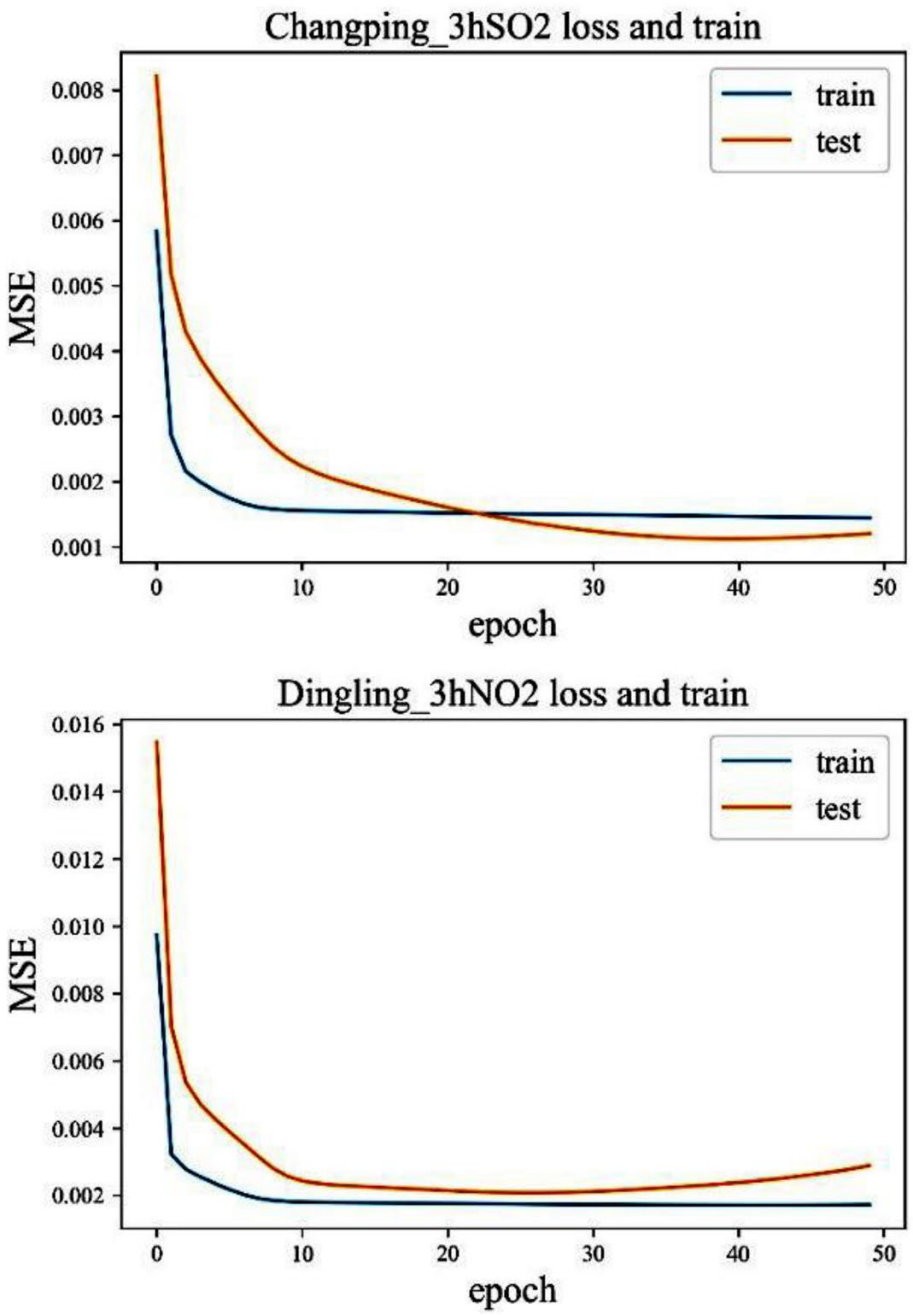
Error trends of training and test sets for the two sites at epoch 50.

The third step was to train the data to adapt to the network, which involved two parameters, batch and epoch. All training samples were divided into several subsets. After all the samples in each subset were finished, the weight parameter would be updated once. The number of samples in this subset was called the batch size, which was set to 72 based on experience. The operation of training all subsets once and updating all gradients was called an epoch. We used four different times of 100, 50, 20, 10 for testing, and compared them with the MSE of the verification data set. It turned out that when the number of training cycles for all samples was 50, the loss function values of the two would overlap earlier (Fig. 6). After the coincidence, over-fitting phenomenon or reverse increase of the error may occur (Fig. 6). When epochs are equal to 20, most stations tend to converge around 20. The final number of iterations for each station adjusted according to this error curve.

In the last step, the test data was substituted into the trained model for prediction, and the final prediction effect was obtained through error evaluation.

### PM_2.5_ predictor structure

#### Outlier handling

When the pollutant concentration was hourly predicted, the existence of outlier will have an important impact on the accuracy of the prediction. Therefore, the main steps of data cleaning for pollutant data were as follows:

The names of 34 stations in the data was obtained, which were used to calculate the missing data and outlier of each station in a loop; All days of the year and all hours of each day from the time series were obtained and stored for missing data interpolation and outlier judgment;

Two new empty arrays were created. One was used to store the time, with the same start and end time as that of the original time column. Its step length is one hour, ensuring the continuous output time. The other array had a length of 24*366 rows, and the number of columns was two fewer than that of original columns, which was used to record the data value corresponding to the moment;

Regarding all column data at a certain time, all data within one day before and after the current data value was firstly selected for judgment. If there were more than half of the missing data on the previous day or the next day, this time would be skipped. If there were four consecutive days of missing data, this time would also be skipped. If neither, the index would be recorded at that moment. Afterwards, whether the data was a null value was judged column by column. If it was a null value, other time would be replenished in line with the above filling method. If it was not a null value, whether it was an outlier would be determined in the interquartile method. The interquartile method is a statistical analysis method. It arranges all the values from small to large and divides them into four equal parts, which are located at three dividing points. Should it be marked as an outlier, then the value would be reset to empty and calculated as missing data.

When a moment was completed, the output file was written in the order of time, station, and concentration of PM_2.5_.

The data at the next moment would be sequentially judged until the last moment, looping through all the time data of this station.

The rest of the station data would be judged in the same method in turn, until all the data was completed, the output file would be saved and ended.

#### Time feature processing

In addition to meteorological conditions that affect the formation and diffusion of pollutants, traffic sources and human activities are also factors that affect the concentration of pollution. The pollution in different time periods is related to the frequency of travel on the day. Therefore, this study analyzed the characteristics of each time, indirectly indicating the intensity of human activities and traffic conditions that day.

Seven categories of statistics are made for each time in the weather data and pollutant data, which were holidays, working days, weekends, the first day of working days, the last day of working days, the first day of rest days and the last days of rest days. Weekends are easier to locate. We directly used the weekday function to perform weekly statistics on the current time. If the result was 5 and 6, it meant Saturday and Sunday. The categories of holidays were also easy to find. We stored all statutory holidays in an array "which_holiday". If it was in the array in turn, we would mark it as 1, otherwise mark it as 0. Working days needs to remove the statutory holidays from Monday to Friday, and then add the days we worked on Saturday and Sunday. Therefore, it was necessary to store the time to work on weekends in an array separately as "which_work". If the result processed by the weekday function was less than 5, and it was not in the array "which_holiday" but in the "which_work", it would be marked as 1, otherwise it was marked as 0. The same method was used to process the remaining four categories. Finally, each day from January 1, 2018 to October 2, 2020 was classified according to the above-mentioned category features, and 7 new feature columns were obtained.

#### Station matching

In addition, weather stations and air quality stations should match with each other. By importing the latitude and longitude of the two into ArcMap software, the neighboring stations were matched through the shortest distance. The matching results were shown in Table 6 below, which were stored in the same table.

**Table 6:** Matching results of the weather station and air quality station.

ID	Air quality station	Weather station	Type
1	Fangshan	Fangshan	Suburbs
2	Daxing	Daxing	
3	Yizhuang	Beijing	
4	Tongzhou	Tongzhou	
5	Shunyi	Shunyi	
6	Changping	Changping	
7	Mentougou	Mentougou	
8	Pinggu	Pinggu	
9	Huairou	Huairou	
10	Miyun	Miyun	
11	Yanqing	Yanqing	
12	Dongsi	Chaoyang	Six major urban areas
13	Tiantan	Beijing	
14	Guanyuan	Haidian	
15	Wanshouxigong	Fengtai	
16	Aotizhongxin	Chaoyang	
17	Nongzhanguan	Chaoyang	
18	Wanliu	Haidian	
19	Beibuxinqu	Haidian	
20	Fengtai Garden	Fengtai	
21	Yungang	Fengtai	
22	Gucheng	Shijingshan	
23	Dingling	Changping	Contrast point and area point
24	Badaling	Yanqing	
25	Miyun reservoir	Shangdianzi	
26	Donggao Village	Pinggu	
27	Yonglidian	Tongzhou	
28	Yufa	Daxing	
29	Liuli River	Fangshan	
30	Qianmen	Beijing	Traffic pollution monitoring point
31	Yongdingmennei	Beijing	
32	Xizhimenbei	Haidian	
33	Nansanhuan	Fengtai	
34	Dongsihuan	Chaoyang	

The matching process was introduced as follows. First, the names of all air quality stations were matched to the corresponding names of the weather stations in turn to obtain an initial matching station data. Then, 24 stations that did not have a corresponding name were saved as a list and matched according to the rules in Table 6. For example, the Olympic Sports Center, East Fourth, East Fourth Ring and Agricultural Exhibition Hall, which were all situated in Chaoyang District, were stored in one list. After that, a new table named “match” was created to store the wind speed and direction of weather stations in Chaoyang District. When the name of the air quality station was consistent with the name in the list, name of this station was changed to the station name same as the one in the list and appended to the original matching station data. The above operations were carried out in turn until all stations were matched. After the space stations were matched, the time of two data sets "station_id" and "UTC_time" would be automatically matched in the merge function. Finally, the output data after space–time matching was obtained.

**Figure 7:**
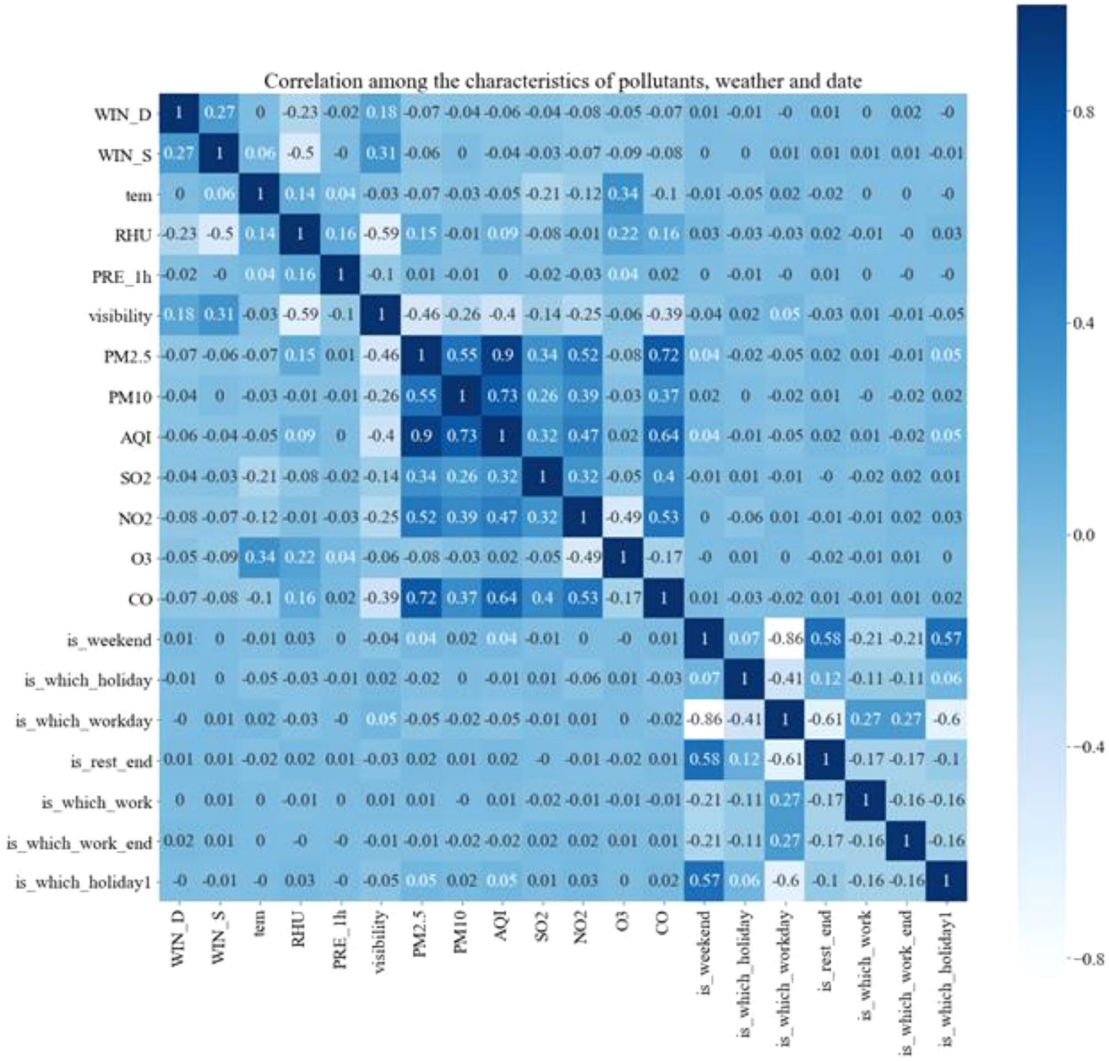
Correlation between pollutant data and input factors after spatio-temporal matching.

After matching the meteorological data and pollutant data and time characteristics of each station, the correlation results among them were shown in Fig. 7. It can be seen from the figure that the relative humidity of meteorological data was negatively correlated with visibility. The positive correlation between AQI (Air Quality Index) and the concentration of PM_2.5_ in pollutant data was the strongest and reached 0.9. The primary factor affecting air quality was still PM_2.5_, followed by PM_10_, which was less than 0.1. In addition, it can also be seen that the meteorological factor that had a greater correlation with PM_2.5_ was visibility. In terms of time characteristics, the negative correlation between weekends and working days was the largest. Through the correlation analysis among the various factors, it can be concluded that the factors that affect the concentration of pollutants selected in this study were representative and had small overlap. At the same time, we acquired a certain understanding of the relationship among the various characteristics.

## Results and discussion

### Comparison of the proposed PM predictor with LightGBM prediction methods

After the predictions of two models, the final prediction results were compared and displayed using the RMSE evaluation index. Finally, Yizhuang was selected as the suburbs, Guanyuan as the main urban area, Yufa as the control area, and East Fourth Ring Road as the traffic station, to display the time series predictions.

#### Prediction results and accuracy of LightGBM model at all stations

First, data of all stations were integrated into the same model for training, and three different input data types resulted in different prediction results. The accuracy statistics were shown in Table 7 below.

**Table 7:** Comparison of prediction results of three pollutant parameters in LightGBM model for all sites.

	RMSE_PM_2.5_		
	Full normalization	Not normalized	Input normalization
East fourth ring	31.396	33.12	32.45
Guanyuan	32.20	28.68	28.10
Yizhuang	31.07	31.84	30.38
Yufa	42.99	38.09	35.88

It can be seen from the table that when the input factors were normalized, the predicted result of PM_2.5_ concentration was better than the unnormalized one, and the RMSE was smaller, which indicating that the factors with different dimensions that were input at the same time has a certain influence on the output results. When the input factors and labels were all normalized, but extreme value range of the training data was denormalized, the prediction results were more polarized. Some results were worse than when they were not normalized, such as Yufa's PM_2.5_ prediction results. Sometimes, normalization on all data were better than just normalizing the input factors, such as the PM_2.5_ prediction result of East Fourth Ring Road. The reason was because the extreme value range of the training data was not exactly the same as the prediction data period.

#### Prediction results and accuracy of lightgbm model at one single station

In order to verify the impact of one model at all stations and one model at one stations on PM_2.5_ prediction results, 34 stations were trained separately, and the prediction results were analyzed. The accuracy indicators were shown in Table 8 below. Since the results of normalizing the labels were not ideal, we did not test this type of input data here. The training results of one model at one station were still consistent with the training results of one model at all stations. When the input factors were normalized, the error of the results was smaller than that of the unnormalized one, which illustrated the importance of the normalization parameter selection.

**Table 8:** Comparison of prediction results of three pollutant parameters in LightGBM model at a single site.

	RMSE_PM_2.5_	
	Unnormalized	Input normalization
Dongsihuan	32.57	30.99
Guanyuan	34.58	34.14
Yizhuang	32.17	29.91
Yufa	42.29	41.01

Comparing Tables 7 and 8, we can see that the impact of the model parameter on the accuracy improvement was not as large as the normalization of the input factor, and even results of one model at one station were not as accurate as those of one model at all stations, such as the prediction of PM_2.5_ at Guanyuan Station and Yufa station. When the data of all stations was placed in one model, the amount of data participated in training was larger than that of placing date of one station in one model, which exactly reflected the superiority of machine learning for massive data analysis. As long as the amount of input data was large enough, the model prediction results will generally be more accurate. As a result, when the decision tree model was used to predict the concentration of air pollutants, the input factors should be labeled and put in a model for training. The model should be unified, but the operating speed should be optimized, for it took a long time to debug the parameters when the amount of data was large.

### LSTM prediction results and evaluation

#### LSTM 3-h input prediction results and accuracy evaluation

The comparison of only normalization on the input factors, normalization on all date and denormalization on all data in the LightGBM model showed that only when the input factors were normalized, the prediction results were better. Therefore, the same data input method was adopted in the training process of the LSTM model, and no attempt was made on the other two types of data. Meanwhile, the accuracy of one model at one station and one model at one station in the LightGBM model was not very noteworthy, which therefore will not be compared again here. Data of one station was used to predict the PM_2.5_ pollutant concentration. As the input of different durations will have a certain impact on the output, the 3-h input and the 12-h input were selected to determine the influence in different time periods. This section mainly demonstrated the prediction results and accuracy evaluation of the 3-h input. The parameters of the were different for each training, so the prediction results were also uncertain. Generally, data in neural network model required multiple trainings. This article has conducted three trainings for each station. The prediction accuracy results were counted in Table 9, which showed the prediction effect of LSTM was significantly better than that of the LightGBM model, and the error was obviously lower by half. Air quality stations and corresponding weather stations are shown in Table 9.

**Table 9:** PM_2.5_ prediction accuracy of LSTM model for air quality stations and its corresponding weather stations.

ID	Air quality stations	RMSE	Weather stations	Type
1	Fangshan	9.334	Fangshan	Suburbs
2	Daxing	7.401	Daying	
3	Yizhuang	7.913	Beijing	
4	Tongzhou	9.392	Tongzhou	
5	Shunyi	7.865	Shunyi	
6	Changping	10.634	Changping	
7	Mentougou	9.572	Mentougou	
8	Pinggu	8.091	Pinggu	
9	Huairou	8.363	Huairou	
10	Miyun	8.695	Miyun	
11	Yanqing	11.85	Yanqing	
12	Dongsi	9.248	Chaoyang	Six major urban areas
13	Tiantan	13.443	Beijing	
14	Guanyuan	9.694	Haidian	
15	Wanshouxigong	10.061	Fengtai	
16	Aotizhongxin	10.410	Chaoyang	
17	Nongzhanguan	8.63	Chaoyang	
18	Wanliu	10.768	Haidian	
19	Beibuxinqu	11.103	Haidian	
20	Fengtaihuayuan	11.59	Fengtai	
21	Yungang	10.014	Fengtai	
22	Gucheng	11.486	Shijingshan	
23	Dingling	8.732	Changping	Control Points and Regional Points
24	Badaling	11.238	Yanqing	
25	Miyunshuiku	6.199	Shangdianzi	
26	Donggaocun	9.098	Pinggu	
27	Yongledian	13.221	Tongzhou	
28	Yufa	13.234	Daying	
29	Bolihe	12.842	Fangshan	
30	Qianmen	8.597	Beijing	Traffic pollution monitoring point
31	Yongdingmennei	10.509	Beijing	
32	Xizhimenbei	13.559	Haidian	
33	Nansanhuan	10.014	Fengtai	
34	Dongsihuan	13.862	Chaoyang	

Among all the stations, the Miyun Reservoir owned the smallest PM_2.5_ prediction error. The maximum RMSE of the East Fourth Ring Road was 13.862, which was much lower than the result predicted by LightGBM, namely 30.99. At the same time, it can be seen that the prediction error of the suburbs was significantly lower than that of the main urban area and traffic pollution points, because there were few meteorological stations in the main urban area. As there was no weather station in the Dongcheng and Xicheng Districts, there will be a certain error in replacing it with adjacent weather conditions. Certainly, it may also be related to the PM_2.5_ value range of the station.

**Figure 8:**
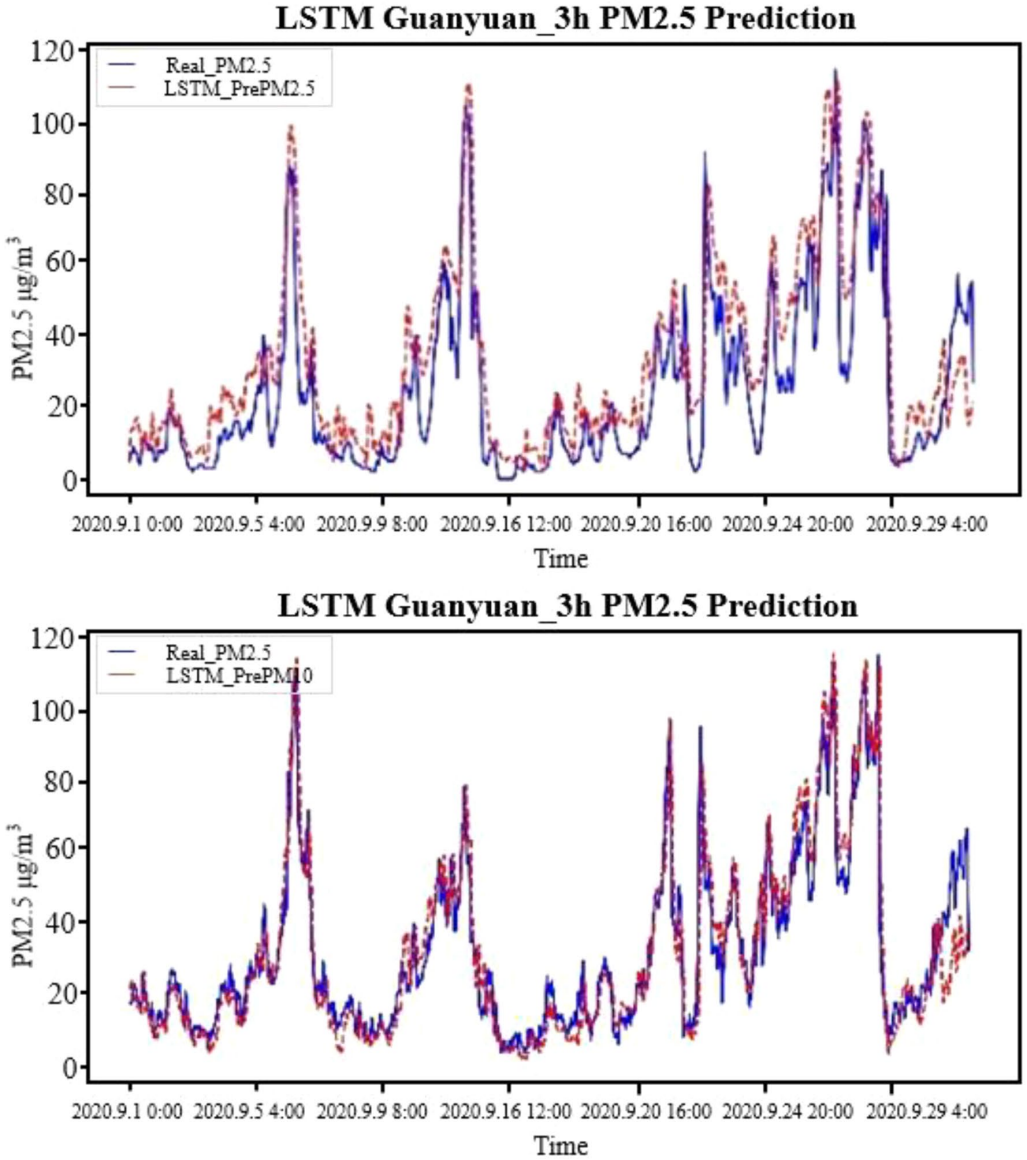
LSTM 3-h input factor normalized PM_2.5_ pollutant prediction fitting results.

To better compare the fitting effects of the LSTM model and the LightGBM model, the same four stations were selected for display. The fitting result of the prediction result and the true value was shown in Fig. 8, which proved that although there were some high values, on the whole, the difference between the predicted result and the true value was smaller, and the trend of change was consistent.

#### LSTM 12-h input prediction results and accuracy evaluation

**Figure.9:**
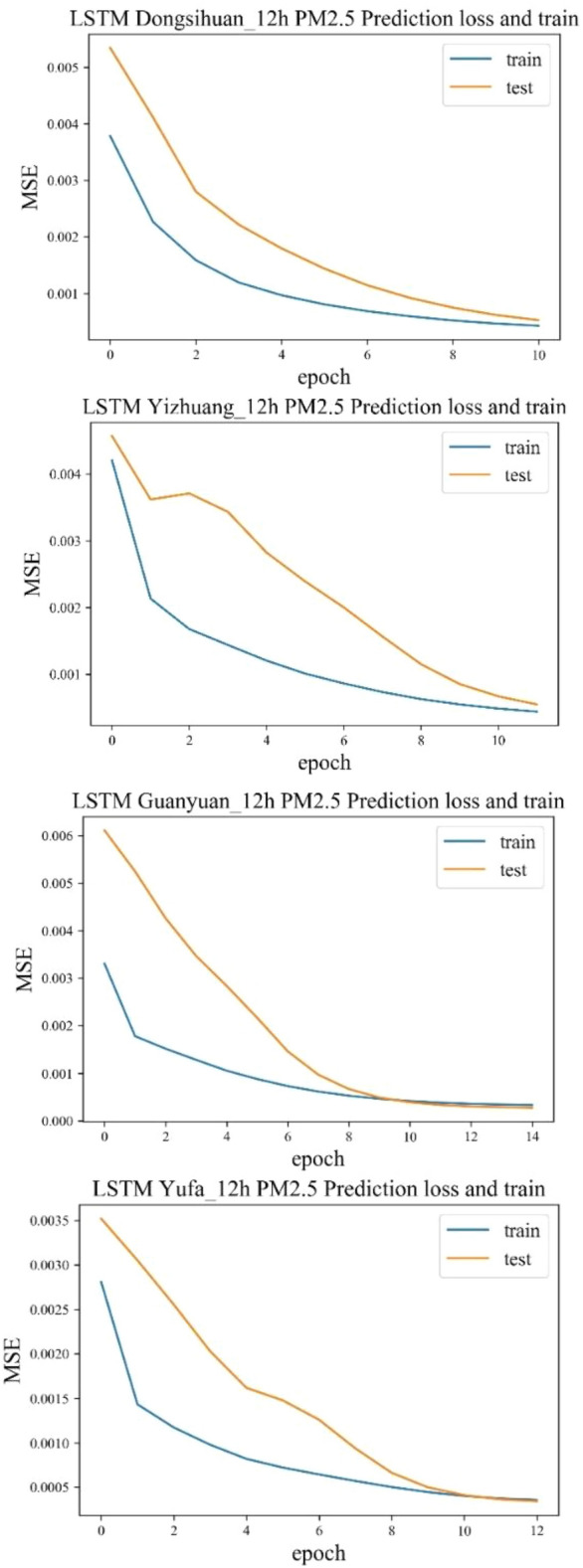
Error comparison between LSTM 12-h input PM_2.5_ training set and test set.

Through the error analysis of the PM_2.5_ data training of the selected four stations, it was found that when the Epoch was around 10, the model converged, and the prediction result at this time was the best. If the error of the test set was lower than that of the training set, overfitting would occur. Generally, the errors of the test set and the training set were intersected or both were stable at one value. When both were in a horizontal state, the model had converged. The four graphs shown in Fig. 9 were all convergent. Under the condition of model convergence, the accuracy of the pollutant prediction results of the four stations and the accuracy results of 3 h were compared (see as Fig. 9). The 12-h LSTM pollutant concentration prediction results were still much better than those of the LightGBM model. But, compared with the 3-h prediction result, the PM_2.5_ of the East Fourth Ring Road had been changed to 12 h, and the RMSE was slightly reduced. However, increase of the input information does not improve accuracy of all stations. The length of input time should be determined based on the training data. Only through multiple trainings can you find a suitable time length.

This article introduced the principles of two machine learning methods used in time series predictions, namely improved decision tree model and neural network model, providing basic knowledge for understanding the construction process of prediction models. The data processing workflow was subsequently explained. After that, the two types of processed data became continuous in time, but did not overlap in space, which was exhibited by comparing the latitude and longitude of the weather station and the air quality station. Therefore, spatial matching was required. Finally, the processed data was added with time characteristics and correlation analysis.

**Figure 10:**
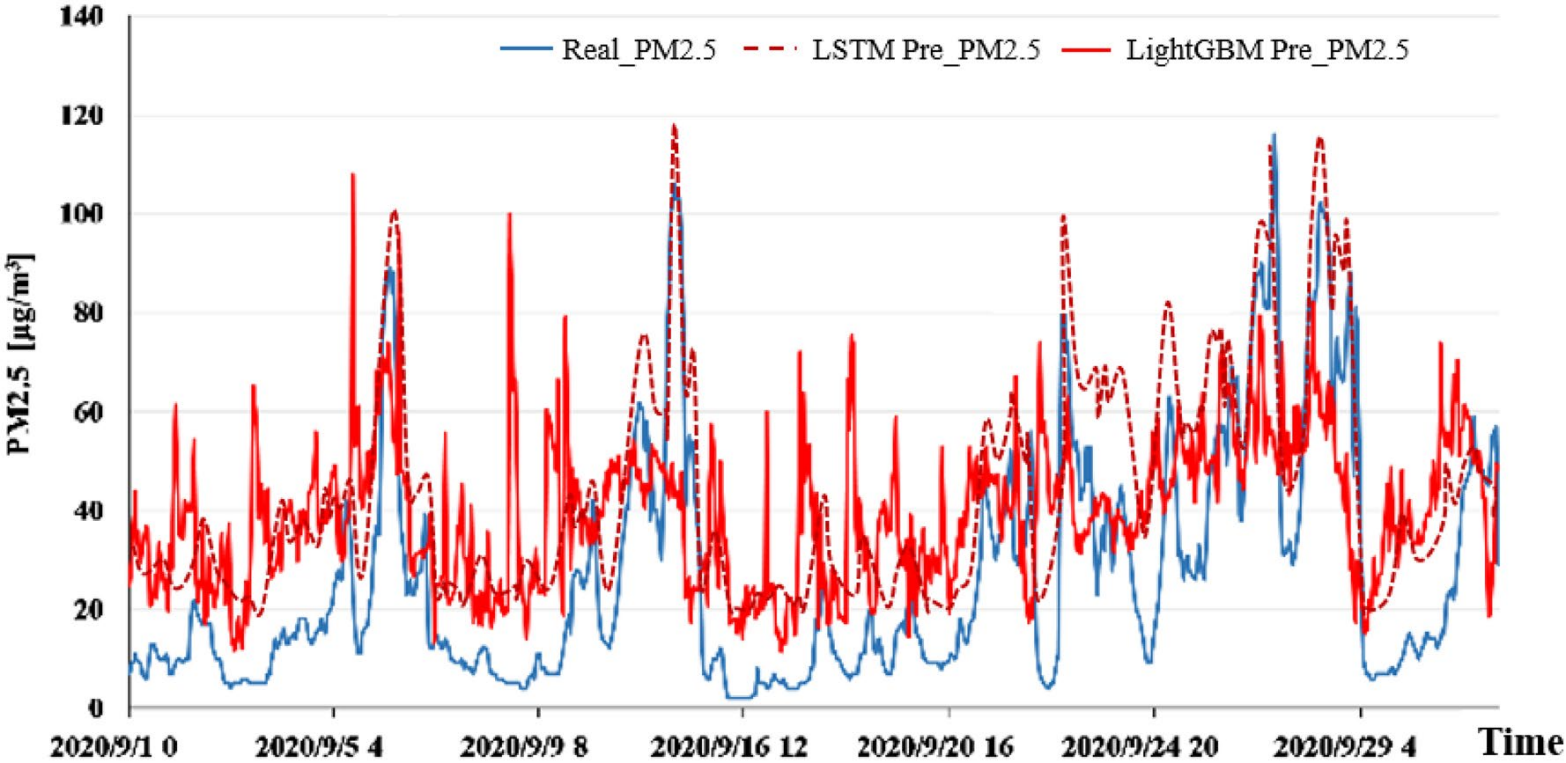
Comparison of prediction results between LightGBM and LSTM models at Dongsi air quality station.

In the process of model construction, because the dimensions of each column of meteorological data and pollutant data were not the same, the input data was divided into three states, namely, denormalization on all data, normalization only on input factors and normalization on all data, so as to verify the impact of data dimensions on the prediction model. Comparing the predicted RMSE in each case, it turned out that the results of normalization on all data were the worst, and the results of normalization only on input factors were the best. Thus, the data that only normalizes the meteorological conditions and time characteristics were finally selected to participate in model training. In addition, a comparative analysis of training data at 34 stations in a unified model and training data at one station in a model was conducted via the LightGBM prediction model, which testified that the difference between the two was not very obvious. Thus, comparison of all-site and single-site models was not performed in the neural network model. The comparison between the prediction results of LightGBM and LSTM models at Dongsi air quality station is shown in Fig. 10.

Different input steps were set in the prediction results of the neural network model, because it add historical pollutant data compared with the decision tree model. However, it was necessary to explore how long the input of historical data was most beneficial to the prediction results. After comparing the results of three-hour and 12-h data, this study concluded that the prediction result of 3 h was better than 12 h most of the time. Thus, 3 h were used as the final input data of the LSTM prediction model.

Through the prediction accuracy and fitting curve analysis of these two models, it can be seen that the effect of LSTM was significantly better than that of LightGBM model, and the RMSE was reduced by nearly half. Yi Ting Tsai et al. Used LSTM to predict the predicted value of hourly PM_2.5_ concentration in Taiwan, and its accuracy can reach a high level^48^. This study has been similarly verified in Beijing.

## Conclusion

By comparing the prediction results of the two models, it was found that the RMSE of each pollutant predicted by the LSTM model was nearly 50% lower than that of LightGBM, which was also more consistent with the fitting curve between actual observations. Exploration on the input step size of the LSTM model expressed that the accuracy of the 3-h input data was higher than that of 12-h input data. Prediction models required pre-process of the input data, including input feature extraction, input factor normalization, and data outlier processing. In addition, training data of all stations in one model had little improvement in accuracy compared to training data of one station in one model.

As the impact of air pollution on daily life and people’s health has become more and more prominent, various countries and localities have gradually established a system of multiple monitoring mechanisms and accumulated massive amounts of historical pollution data. Since station monitoring is concentrated on points, it is possible to develop remote sensing data and site data fusion prediction research. Station monitoring has the characteristics of high frequency of all-day monitoring, large area of remote sensing, but lack of evening data. We should comprehensively study the advantages of various monitoring methods and avoid shortcomings, so as to obtain prediction data with high temporal and spatial accuracy and protect people's lives and health.

## Supplementary Information

Supplementary Information.

## Data Availability

All data generated or analysed during this study are included in this published article [and its supplementary information files].
